# Phase II and pharmacological study of oral paclitaxel (Paxoral) plus ciclosporin in anthracycline-pretreated metastatic breast cancer

**DOI:** 10.1038/sj.bjc.6603332

**Published:** 2006-09-12

**Authors:** H H Helgason, C M F Kruijtzer, A D R Huitema, S G Marcus, W W ten Bokkel Huinink, M E Schot, J H Schornagel, J H Beijnen, J H M Schellens

**Affiliations:** 1 Department of Medical Oncology, The Netherlands Cancer Institute/Antoni van Leeuwenhoek Hospital, Plesmanlaan 121, 1066CX Amsterdam, The Netherlands; 2 Rivierenland Hospital, Tiel, The Netherlands; 3 Slotervaart Hospital, Louwesweg 6, 1066EC Amsterdam, The Netherlands; 4 IVAX Research Inc., Miami, FL, USA; 5 Faculty of Pharmaceutical Sciences, Utrecht University, Utrecht, The Netherlands

**Keywords:** phase II, paxoral, advanced breast cancer

## Abstract

Paclitaxel is an important chemotherapeutic agent for breast cancer. Paclitaxel has high affinity for the P-glycoprotein (P-gp) (drug efflux pump) in the gastrointestinal tract causing low and variable oral bioavailability. Previously, we demonstrated that oral paclitaxel plus the P-gp inhibitor ciclosporin (CsA) is safe and results in adequate exposure to paclitaxel. This study evaluates the activity, toxicity and pharmacokinetics of paclitaxel combined with CsA in breast cancer patients. Patients with measurable metastatic breast cancer were given oral paclitaxel 90 mg m^−2^ combined with CsA 10 mg kg^−1^ (30 min prior to each paclitaxel administration) twice on one day, each week. Twenty-nine patients with a median age of 50 years were entered. All patients had received prior treatments, 25 had received prior anthracycline-containing chemotherapy and 19 had three or more metastatic sites. Total number of weekly administrations was 442 (median: 15/patient) and dose intensity of 97 mg m^−2^ week^−1^. Most patients needed treatment delay and 17 patients needed dose reductions. In intention to treat analysis, the overall response rate was 52%, the median time to progression was 6.5 months and overall survival was 16 months. The pharmacokinetics revealed moderate inter- and low intrapatient variability. Weekly oral paclitaxel, combined with CsA, is active in patients with advanced breast cancer.

As the incidence of breast cancer rises with advancing age, the total number of women with breast cancer will increase (Feigelson *et al*, 1997). Paclitaxel is widely used in advanced breast, lung and ovarian cancer. In breast cancer, paclitaxel has demonstrated activity as a single agent in first- and second-line treatment and in combination schedules (Van Poznak *et al*, 2002). Tumour response rates of 20–30% have been observed in several phase III studies in second-line treatment (Di Leo and Piccart, 1999). Weekly infusions of paclitaxel have gained wide popularity because of the favourable toxicity profile allowing dose intensifications (Seidman *et al*, 1997; Perez *et al*, 2001). Seidman *et al* (1997) reported in a phase II study using weekly 100 mg m^−2^ an overall response rate (ORR) of 53% in 30 breast cancer patients who had failed first-line chemotherapy (Perez *et al*, 2001). Therapy was well tolerated and remarkable for a lack of overall and cumulative myelosuppression. Grade 3 sensory neuropathy was observed in 10% of patients receiving doses of less than 100 mg m^−2^ per weekly cycle (Seidman *et al*, 1997). Other phase II studies have confirmed response rates between 50 and 68% and a favourable safety profile of doses in the range of 80–100 mg m^−2^ per weekly cycle in first-line treatment of advanced breast cancer (Bernard-Marty *et al*, 2003; Nisman *et al*, 2003; Sato *et al*, 2003; Sledge *et al*, 2003; Toyama *et al*, 2003). However, it remains controversial what the optimal dose and schedule is. Sikov *et al* recently reported that a weekly schedule of 80 mg m^−2^ intravenous (i.v.) induced less toxicity than higher weekly doses of 150 mg m^−2^ weekly × 6 q8wks and 175 mg m^−2^ weekly × 2 q3wks (Green *et al*, 2005). There is also evidence that weekly paclitaxel can be effective as rescue in patients who have failed paclitaxel administered every 3 weeks (Perez *et al*, 2001).

Oral administration of paclitaxel is convenient and practical for patients and circumvents systemic exposure to the vehicle Cremophor EL, which is held responsible for hypersensitivity reactions (Meerum Terwogt *et al*, 1998). Moreover, oral administration enables the development of chronic treatment schedules, resulting in sustained plasma concentrations above a pharmacologically relevant threshold level. Preclinical studies have shown that oral bioavailability of paclitaxel is low due to its affinity for the membrane-bound drug efflux pump P-glycoprotein (P-gp) in the gastrointestinal tract. The primary routes of paclitaxel presystemic extraction and elimination consist of successive hydroxylation reactions mediated by the cytochrome *P*450 CYP2C8 (mainly), CYP3A4 and CYP3A5 isoforms (Sparreboom *et al*, 1997; Sparreboom and Verweij, 2003). Preclinical and clinical studies in our institute have shown that coadministration of oral ciclosporin (CsA), an efficacious inhibitor of P-gp as well as of CYP3A4-mediated drug metabolism, results in an approximately eightfold increase in the systemic exposure to oral paclitaxel. Inhibition of CYP3A4 may play a role in the increased bioavailability of paclitaxel, but the bioavailability increased from 4% without CsA to 47% with CsA (Meerum Terwogt *et al*, 1998; van Asperen *et al*, 1998; Meerum Terwogt *et al*, 1999). Another study revealed that P-gp inhibition by CsA was maximal at a single dose of 10 mg kg^−1^ (Malingre *et al*, 2001b). Other paclitaxel formulation, based on an albumin-stabilised nanoparticle, has also the ability to circumvent the systemic exposure to Cremophor EL. This compound, Abraxane®, is effective in anthracycline-pretreated metastatic breast cancer patients and has been approved by the FDA for this indication (www.fda.gov/cder/foi/label/2005/021660lbl.pdf).

In order to improve and prolong the systemic exposure to oral paclitaxel, a two administrations on one-day schedule was investigated in a phase I study. We demonstrated that oral paclitaxel, at the dose level of 90 mg m^−2^ twice, reached the highest systemic exposure with a good safety profile (Malingre *et al*, 2001a). With these issues in mind, we initiated a phase II and pharmacological study with the combination of oral paclitaxel and oral CsA twice on one day each week in patients with advanced breast cancer, previously treated with anthracycline-containing chemotherapy. Our primary objective was to evaluate the antitumour activity of this schedule, measured by frequency of objective response and time to progression, in patients with recurrent or metastatic breast cancer. The secondary objectives were the toxicity, overall survival and the pharmacokinetics of the combination.

## PATIENTS AND METHODS

### Eligibility criteria

Patients with histologically confirmed, advanced breast cancer were eligible for this study. Prior chemotherapy, radiotherapy or hormonal therapy was allowed, but had to be discontinued for at least 4 weeks prior to study entry. All patients had to have WHO performance status ⩽2 and should have received a prior anthracycline-containing chemotherapy. A maximum of two prior chemotherapy regimens, one of which given for metastatic disease, was allowed. Prior taxane therapy was not allowed. At entry, patients were required to have measurable disease according to the RECIST criteria (Tsuchida and Therasse, 2001). They had to have adequate haematological, renal and hepatic functions (absolute neutrophil count (ANC)>1.5 × 10^9^ l^−1^, platelets>100 × 10^9^ l^−1^, bilirubin⩽1.5 times upper limit of normal (ULN), AST and/or ALT ⩽2.0 ULN, but in the presence of liver metastases ⩽5.0 ULN; serum creatinine ⩽2.0 times ULN). Exclusion criteria were as follows: concomitant use of known P-gp inhibitors and chronic use of H_2_-receptor antagonists or proton pump inhibitors; known history of cerebral or leptomeningeal metastases; history of prior malignancy, except completely excised *in situ* carcinoma of the cervix or nonmelanoma skin cancer; bowel obstruction or motility disorders that could influence the absorption of drugs; concurrent treatment with other experimental drugs; allergy to CsA; concomitant medication which has been reported to increase the metabolism of CsA; serious concurrent disease; unresolved toxicities of previous treatment (⩾grade 2); angina or myocardial infarction in the 6 months prior to study entry; and second or third degree AV block without pacemaker, or congestive heart failure. The study protocol was approved by the Medical Ethics Committees of all five participating institutes (see Acknowledgments) and all patients gave written informed consent.

### Treatment plan

On day 1 of each week, oral paclitaxel (Paxoral®, IVAX research, Inc. Miami, USA) was administrated twice (90 mg m^−2^ × 2) with at least seven, but not more than 12 h dose interval. Ciclosporin in a dose of 10 mg kg^−1^ was given 30 min prior to each dose of oral paclitaxel. Ciclosporin (Neoral®, Novartis, Basel, Switzerland) was supplied as capsules of 50 and 100 mg, or as a liquid solution of 100 mg ml^−1^. Oral paclitaxel was supplied as a solution of 12 mg ml^−1^ in a bottle. For further information about the oral administration and dietary advise, see our previous publication (Kruijtzer *et al*, 2003). Our previous phase I studies revealed that antiallergic premedication (dexamethasone, clemastine, ranitidine) could be omitted (Malingre *et al*, 2000). Only oral granisetron was given 1 h prior to intake of the chemotherapy to prevent nausea and vomiting. This treatment was administered weekly until disease progression or unacceptable toxicity developed.

### Evaluation of response

Standard clinical measurements and radiological examinations were used to ensure measurable disease according to RECIST (Tsuchida and Therasse, 2001) criteria for response evaluation. Radiologic responses were confirmed by independent external radiologic reviews. Patients who completed at least 6 weekly cycles were considered evaluable for response. The primary end point of the study was response rate. We also determined the duration of response, which was defined as the number of days between the onset of response and the date of last progression-free evaluation. Response was determined every 6 weeks. For patients who had not progressed the date of last progression-free evaluation would be censored. We determined the time to progression as the number of days between the date of first treatment and the date on which progression was clearly documented, or death had occurred.

### Evaluation of toxicity

Pretreatment evaluation included a complete medical history and complete physical examination. Haematology and blood chemistries were checked prior to treatment and subsequently weekly. All toxicities were weekly graded according to the National Cancer Institute Common Toxicity Criteria (NCI-CTC, v.2.0 http://ctep.cancer.gov). All patients who received at least one weekly cycle of therapy were evaluable for toxicity. In case of toxicity, two dose reductions were allowed: first to 70 mg m^−2^ (twice) and then to 55 mg m^−2^ (twice) or 90 mg m^−2^ (once) if the patient experienced persistent nausea or vomiting after the morning administration. Patients, who required further dose reductions, were withdrawn from the study. Nonhaematological toxicity of grade 3 or 4 (except inadequately treated nausea and vomiting) or haematological toxicity consisting of ANC of <0.5 × 10^9^ l^−1^, and neutropenic fever or thrombocytopenia <25 × 10^9^ l^−1^ was mandated for dose reduction of one dose level. For patients who required dose reductions, the dosage was not re-escalated in subsequent cycles. Treatment was postponed until recovery of thrombocytes >100 × 10^9^ l^−1^ and neutrophils >1.5 × 10^9^ l^−1^. The CsA dose of 10 mg kg^−1^ remained constant.

### Sample collection and analysis

Pharmacokinetic monitoring was performed during the first two weekly cycles. For paclitaxel, blood samples of 5 ml were collected in heparinised tubes, at 0, 30 and 60 min and at 2, 3, 4, 6, 7, 7.5, 8, 10, 12, 24 and 30 h after ingestion of paclitaxel. For further information on sample collection and paclitaxel analysis, see previous publication (Kruijtzer *et al*, 2003).

### Pharmacokinetic analysis

A population pharmacokinetic model was developed for paclitaxel by using the nonlinear mixed-effect modelling program NONMEM (double precision; version V, level 1.1) as published previously (Kruijtzer *et al*, 2003). Briefly, the first-order conditional estimation method was applied. A two-compartment structural kinetic model with first-order absorption and elimination and saturable transport between central and peripheral compartment was used to describe the time profiles of paclitaxel plasma concentration. The pharmacokinetics of paclitaxel were parameterised in terms of absorption rate constant (*K*_a_), volume of distribution of the central compartment (*V*), clearance from the central compartment (CL), maximal transport capacity from the central to the peripheral compartment (TR_max_), the concentration at which the transport is half-maximal (TR_m_) and rate constant for transport form the peripheral to the central compartment (*k*_21_). Since paclitaxel was administered orally, the plasma terms ‘volume of distribution’, ‘clearance’ and ‘maximal transport capacity’ represent the ratio of these parameters (*V*, CL, TR_max_) to the unknown bioavailability.

Individual pharmacokinetic parameters were estimated by Bayesian approach. On the basis of these parameters, individual plasma concentration–time profiles were generated for the assessment of the area under the plasma concentration–time curve (AUC), the maximal plasma concentration (*C*_max_), the time to maximal plasma concentration (*T*_max_) and the time above the previously defined threshold concentrations of 0.1 *μ*M (*T*>0.1 *μ*M) and 0.05 *μ*M (*T*>0.05 *μ*M). The values of *T*_max_ and *C*_max_ were directly determined from the experimental data. For CsA, the AUC was determined according to the trapezoidal method by Pharsight® WinNonlin™, ed. 5.0.1.

### Statistics

Patients were accrued according to a two-stage design (Simon, 1989) aiming at 25 eligible patients. Analyses of response rate and time to progression were performed on all evaluable patients and on the total population. The time to progression and overall survival curves were estimated using the Kaplan–Meier method.

## RESULTS

### Patients and characteristics

In total, 29 patients were recruited between November 2000 and February 2003 in four cancer centres (The Netherlands Cancer Institute, Amsterdam, The Netherlands; Innere Universitätsklinik und Poliklinik, Essen, Germany; Kliniken St Antonius, Wuppertal, Germany; and Ashford Cancer Centre, Ashford, Australia (23, 3, 2 and 1 patients, respectively) in this study. Four patients were not eligible: three because of lack of previous anthracycline-containing chemotherapy and one did not meet the RECIST criteria for evaluation. Response evaluation was not formally possible in two patients according to the definition of the protocol, in one patient because of disease progression after two administrations and one patient was lost to follow-up after the first paclitaxel administration. All, except one (lost to follow-up), were eligible for toxicity evaluation. Table 1 lists the clinical characteristics of all patients. Median age was 50 years (range 34–66 years) and all, except one patient, were postmenopausal at the start of study treatment. Eighteen (62%) had oestrogen- and/or progesterone-positive tumour. Twenty-six (90%) patients had more than one metastatic site, mostly bone (69%), liver (62%) and lymph node metastases (55%). Of 23 evaluable patients, nine (39%) received prior adjuvant anthracycline chemotherapy, 14 (61%) received prior palliative anthracycline chemotherapy and three patients received prior two (anthracycline plus CMF (2) or vinorelbine (1)) treatment lines. Of evaluable patients, 15 (65%) received prior antihormonal therapy, five as adjuvant treatment, 10 as palliative (range 1–4) treatment and four patients received both adjuvant and palliative antihormonal therapy.

### Dose intensity and dose reduction

Twenty-nine patients received in total 442 weekly oral paclitaxel and CsA administrations with a median of 15 (range 1–36) per patient. Of those 442 administrations, 299 (68%) were two doses and 143 (32%) were in one dose because of nausea and vomiting in seven patients (24%) after the morning dose. Of the 299 administrations, 191 (64%) were given according to the standard dose of 90 mg m^−2^, and 87 (29%) and 21 (7%) at the reduced dose of 70 and 55 mg m^−2^, respectively. Of the 143 administrations, 87 were given at a dose of 90 mg m^−2^, and 21 and 35 at the reduced dose of 70 and 55 mg m^−2^, respectively. Overall, the median dose intensity was 97 mg m^−2^ week^−1^ (range 52.4–180 mg m^−2^ week^−1^). One patient received only one intake of paclitaxel and refused thereafter further treatment. Seventeen patients needed dose reductions, mostly (59%) because of moderate neutropenia, which did not resolve within 1 week of delay, or nausea and vomiting after the morning intake. Twenty-six patients experienced treatment delay of in total 234 weekly administrations, median 8 (range 0–38), mostly because of grade 3–4 neutropenia.

### Response

Antitumour activity of paclitaxel combined with CsA was evaluable in twenty-three patients (Table 2). There were no complete responses. Partial response, for more than 12 weeks, was seen in 15 patients, resulting in an ORR of 51.7% (95% confidence interval (CI), 33.5–69.9%) in all 29 patients and 65.2% (95% CI, 45.7–84.7%) in all 23 for response-evaluable patients. Four other patients had disease stabilisation, for more than 12 weeks, resulting in meaningful disease stabilisation (partial response or stable disease) in 65.5% of all patients and 82.6% of response-evaluable patients. Four of the response-evaluable patients (17.4%) had progressive disease at the time of first evaluation after 6 weekly administrations.

### Treatment duration

As of December 2003, all patients have discontinued treatment. Of the 15 patients who had an initially confirmed partial response, 10 patients later discontinued treatment as a result of disease progression after, respectively, 25–45 (mean 34) and 14–36 (mean 23) weeks administrations. Four patients discontinued treatment because of toxicity after, respectively, 6, 8, 11 and 28 weekly administrations. One patient was lost to follow-up after 17 weekly administrations. Two patients with disease stabilisation stopped because of disease progression after, respectively, 16 and 18 weeks of treatment. Two other patients stopped because toxicity (grade 4 myelosuppression, grade 2 nausea and vomiting) developed after 16 weeks of treatment and, respectively, 7 and 11 weekly administrations. The patient who stopped because of grade 4 myelosuppression was treated with weekly paclitaxel i.v. for another 12 weeks before disease progression. Four patients stopped after first evaluation after six administrations because of progressive disease. Of the six patients who were not formally evaluable for response, two patients discontinued treatment because of toxicity after, respectively, 13 and 15 weekly administrations, two patients discontinued treatment because of disease progression after, respectively, 2 and 33 weekly administrations, one was lost to follow-up after eight administrations and one refused further treatment after one single intake and is lost to follow-up.

### Time to progression and overall survival

**Figure 1:**
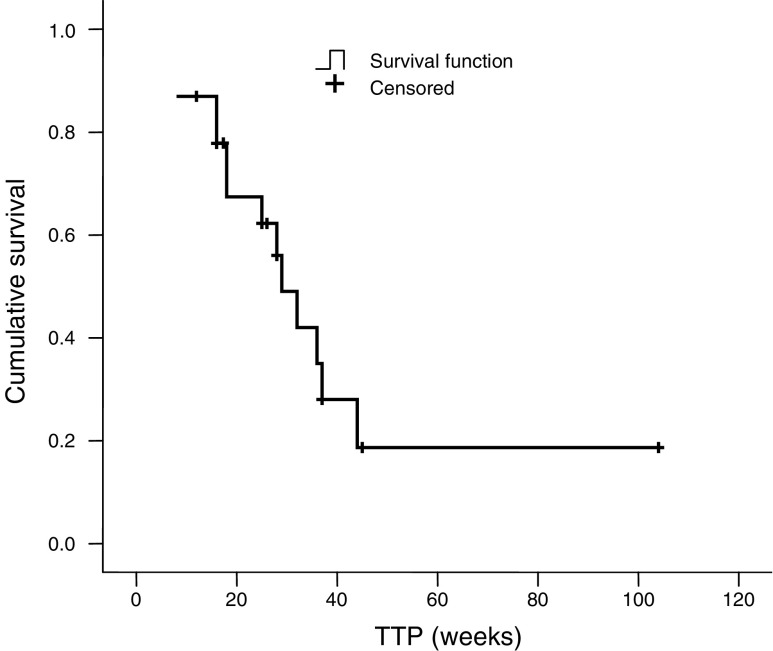
Kaplan–Meier curve of time to progression in evaluable patients (*n*=23).

As of December 2003, the median time to progression was 6.5 months (95% CI, 4–10) in evaluable patients. Of all patients, 16 are deceased, seven are alive at, respectively, 10.5, 12.5, 12.5, 15.5, 29.5, 32 and 36.5 months after starting treatment and six are lost to follow-up after, respectively, 0.5, 2, 2.5, 5.5, 10.5 and 19 months from the start of treatment. Median overall survival was 16 months (95% CI, 9–24) for all patients. The Kaplan–Meier curve of overall survival of all patients is shown in Figure 1.

### Toxicity

All patients were eligible for toxicity evaluation, except one, who was lost to follow-up after one administration. Eight (27.6%) patients had to stop their treatment because of toxicity, mainly neutropenia (CTC grades 3–4) or nausea and vomiting after the morning intake, after 6, 7, 8, 11 (two patients), 13, 15 and 28 weeks, respectively. The principal haematological toxicity was neutropenia, as 15 (54%) patients experienced significant (CTC grades 3–4) neutropenia (Table 3). However, only three events of neutropenic fever and no sepsis were observed. Of the nonhaematological toxicities, gastrointestinal toxicity was most frequently reported (Table 3). Although there were no grade 3 nausea nor grade 3 or 4 vomiting, these toxicities were important reasons for dose reductions and change in dose schedule from twice daily to once daily. Overall grade 1 and 2 nausea and vomiting were reported in 90 and 75% of patients, respectively. Twenty (71%) patients reported diarrhoea, mostly grade 1 and 2, but one patient had diarrhoea grade 3. Serious neurotoxicity (⩾grade 2), as commonly described for treatment with i.v. paclitaxel, was seen in five patients (18%) after an average number of 21 (7–32) administrations. Other toxicities of importance were asthenia in 16 (two grade 3) patients, fatigue in 21 (six grade 3) patients, arthralgia/myalgia in 14 (no grade 3) patients and nail abnormalities in seven patients. Possible because of the short interval between paclitaxel and CsA, we were not able to identify any short-term adverse effects of CsA, but it may have contributed to the diarrhoea and neurotoxicity. Renal toxicity was not observed. There was no grade 4 nonhaematological toxicity and no toxic deaths.

### Pharmacokinetics

Pharmacokinetic analysis was possible in 26 patients covering in total 50 courses of chemotherapy (26 in week 1 and 24 in week 2). Several pharmacokinetic models were applied to the data, including two- and three-compartmental models with linear and/or saturable distribution and/or elimination. The data were best described using a two-compartmental model with first-order absorption, linear elimination and saturable distribution to the peripheral compartment. The value of TR_m_ (the concentration at which the transport rate to the peripheral compartment is half-maximal) could not be estimated and was fixed to a value of 120 *μ*g l^−1^ (0.14 *μ*mol l^−1^) as obtained in a previous analysis on oral application of paclitaxel in patients with gastric cancer (Kruijtzer *et al*, 2003). The model-based and Bayesian-predicted concentrations were symmetrically distributed around the line of identity, indicating the adequacy of the population model (data not shown). On the basis of individual Bayesian estimates, secondary pharmacokinetic parameters of two administrations of oral paclitaxel were derived (Table 4). The mean AUC of orally administered paclitaxel was 4304±1426 *μ*g h l^−1^ (5.04±1.67 *μ*M) in week 1 and 4005±1110 *μ*g h l^−1^ (4.69±1.3 *μ*M) in week 2. The calculated interpatient variability (%CV) of the AUC of paclitaxel was 33.2 and 27.5% in week 1 and 2, respectively. The intrapatient variability (%CV) of the AUC was only 17.4%. The mean *T*>0.1 *μ*M values were 13.2±5.1 h in week 1 and 12.1±3.8 h in week 2. The mean *T*>0.05 *μ*M values were 23.8±8.5 h in week 1 and 22.4±7.5 h in week 2. The mean *C*_max_ values after the first and second dose were comparable in week 1 and 2, but a small increase of the *C*_max_ value was noted after the second dose.

## DISCUSSION

This phase II study with oral paclitaxel combined with CsA, in anthracycline-pretreated women with metastatic breast cancer revealed an ORR of 51.7% and overall survival of 16 months. The ORR in 23 evaluable patients was 65.2% and meaningful disease stabilisation, for more than 12 weeks, was 82.6%. The median time to progression was 6.5 months for all evaluable patients. This response rate lies in the upper range of results of chemotherapy in breast cancer patients (Di Leo and Piccart, 1999; Nisman *et al*, 2003; Lombardi *et al*, 2004b). Three patients were not evaluable because they had not received previous anthracycline-containing chemotherapy. One of these patients achieved partial remission for 17 months and another patient achieved disease stabilisation for 9 months. The third patient was lost to follow-up after eight administrations. Response evaluation was not possible in three patients. It must be realised that the number of evaluable patients was limited and consequently the CI is wide.

The toxicity was manageable and consisted mainly of myelosuppression, which was neutropenia (CTC grade ⩾2 in 68% of patients) without morbidity or mortality, moderate anaemia but no thrombocytopenia. This toxicity pattern is consistent with other reports of i.v. paclitaxel (Seidman *et al*, 1997; Di Leo and Piccart, 1999; Lombardi *et al*, 2004a). Previously, we demonstrated in a phase I study that a relevant systemic exposure of oral paclitaxel, with a good safety profile, was reached at the dose level of 90 mg m^−2^ twice on one day each week (Malingre *et al*, 2001a). The current study supports the moderate toxicity of this regimen. Nausea and vomiting were the most important nonhaematological toxicities and lead most often to omission of the afternoon administration and consequently to alteration in the dose schedule from twice to once daily administration (32% of all weekly administrations). Nausea was more related to the smell and taste of the oral paclitaxel liquid solution than to its emetogenic effects. Recently, we prepared a capsule formulation that may significantly improve gastrointestinal tolerance to oral paclitaxel. Reversible sensory polyneuropathy was seen in 16 patients and was CTC grade 1 in 11 and grade 2 in five patients, respectively. The five patients with CTC grade 2 polyneuropathy received a median of 21 administrations (range 7–32) at a dose intensity of 105 mg m^−2^ week^−1^ and cumulative dose of 3056 mg, suggesting that the cumulative dose of paclitaxel, rather than peak concentration, is the major determinant of this side effect. Cremophor EL is not likely to contribute much to this toxicity as has been shown previously (Mielke *et al*, 2005b). Moderate diarrhoea was reported in 20 patients and was mostly easily manageable.

The mean dose intensity in our study was 97 mg m^−2^ week^−1^ (range 52.4–180 mg m^−2^ week^−1^), or 54% of the planned dose, and the mean number of weekly paclitaxel administrations was 15. This is lower than in our previous reports on weekly oral paclitaxel in gastric (141 mg m^−2^ week^−1^) and non-small-cell lung cancer (172 mg m^−2^ week^−1^) patients (Kruijtzer *et al*, 2002, 2003). This can be explained by the shorter treatment duration related to the shorter time to progression and overall survival in the gastric and lung cancer patients, compared to breast cancer patients (Kruijtzer *et al*, 2002, 2003). The mean dose intensity of 97 mg m^−2^ week^−1^ is higher than reached with the standard 3 weekly i.v. schedule. However, it is comparable with most weekly i.v. paclitaxel schedules of 80–100 mg m^−2^ week^−1^ in patients with advanced breast cancer. Direct comparison of pharmacokinetic values and doses for oral *vs* i.v. paclitaxel must be made with caution, because of the nonlinear pharmacokinetic behaviour of i.v. paclitaxel (Malingre *et al*, 2000), but the incidence of neutropenia supports adequate paclitaxel exposure.

Most patients experienced some form of dose reduction. Twenty-six patients needed a delay of administration with a total 234 delayed weekly cycles of median 8 weeks (range 0–38). This was mostly because of moderate neutropenia, but occasionally on patient request. In total, 29 dose reductions were needed in 17 patients, because of neutropenia or neutropenia and nausea, in 10 and seven patients, respectively. Most of these patients, especially those with nausea, tolerated the once daily dosing better, which enabled further treatment. One patient with stable disease developed grade 4 neutropenia and switched to i.v. paclitaxel for 16 weeks before disease progression. Because of dose reductions approximately 143 administrations (32%) were given once daily. There were no serious adverse reactions or toxic deaths in our study. Future studies need to assess a reduced dose to limit neutropenia allowing continued treatment. Furthermore, should a capsule formulation of paclitaxel lower the incidence of anticipatory nausea and vomiting making the treatment more convenient. Oral administration of paclitaxel circumvents systemic exposure to the vehicle Cremophor EL, which compound is responsible for hypersensitivity reactions, thus enabling us to avoid pretreatment with H_1_ and H_2_ blockers and steroids and their potential adverse effects (Meerum Terwogt *et al*, 1998).

Although we cannot exclude some short-term gastrointestinal adverse effects of CsA, the incidence of nausea was directly related to the smell or taste of paclitaxel. In concordance to our previous results, the weekly dose of CsA was not associated with renal toxicity or infections (Kruijtzer *et al*, 2002, 2003). This can most likely be attributed to the weekly administration of the drug, while after organ transplantation, CsA is administered on a continuous daily basis. At this dose and schedule, CsA is also not expected to have important long-term negative effects.

The pharmacokinetic data indicate good reproducibility of pharmacokinetic parameters of orally administered paclitaxel. The peak plasma concentration was comparable with our previous results (Kruijtzer *et al*, 2002, 2003), but significantly lower than obtained with the conventional three weekly schedule, which might have contributed to the low incidence of neurotoxicity, although the cumulative dose seems to be a major contributing factor to this toxicity (Huizing *et al*, 1993; Mielke *et al*, 2005b). The median time period of paclitaxel plasma concentration above 0.1 *μ*M was 13.2 and 12.1 h in week 1 and 2, respectively, which is comparable to our earlier results (Kruijtzer *et al*, 2002, 2003), but longer than obtained with the conventional three weekly schedule if calculated upon 3-week interval. Population pharmacokinetic analysis revealed that the pharmacokinetics of orally administered paclitaxel were best described by a two-compartmental model with first-order absorption, linear elimination and saturable distribution to peripheral compartment, as has been shown for unbound paclitaxel, which supports the opinion that Cremophor EL is responsible for the nonlinear pharmacokinetics of i.v. paclitaxel (Henningsson *et al*, 2003). The pharmacokinetics of i.v. and by orally administered paclitaxel, with and without Cremophor EL in the systemic circulation, respectively, are substantially different, which makes further comparison of AUC values, obtained after oral and i.v. administration, difficult. The known nonlinear pharmacokinetics of i.v. paclitaxel is due to intravascular paclitaxel entrapment caused by Cremophor EL. Consequently, direct comparison of i.v. and oral paclitaxel could underestimate the true bioavailability of oral paclitaxel (Malingre *et al*, 2001a; Gelderblom *et al*, 2002; Mielke *et al*, 2005a).

The calculated interpatient variability of the AUC of paclitaxel was 33.2 and 27.5% in week 1 and 2, respectively. This variability is moderate and comparable with the variability of other chemotherapy treatments that rely on body surface for dose calculations (Baker *et al*, 2002). The intrapatient variability of the AUC was only 17.4% and indicates a limited variation in the apparent bioavailability of our formulation of oral paclitaxel with CsA.

Use of oral chemotherapy formulations has significant advantages over classical i.v. treatment. It enables outpatient-based treatment, at home, with increased patient comfort and possibly quality of life, but also bi-daily administration resulting in prolonged paclitaxel exposure with potentially higher activity. With the absence of plasma Cremophor EL exposure in oral paclitaxel formulations, we can circumvent the use of antiallergic medications (Meerum Terwogt *et al*, 1998). Abraxane®, an albumin-stabilised nanoparticle formulation of paclitaxel, has also the ability to effectively deliver paclitaxel to the circulation without Cremophor EL, but has the disadvantage of i.v. use.

A disadvantage of oral formulations of chemotherapy is the possible interaction with food and comedication and unpredictable changes in uptake caused by vomiting or diarrhoea. The low intrapatient variability, as observed in current study, shows that this is not a problem in case of oral paclitaxel combined with CsA. The combination is mildly emetogenic and the maximal plasma c is reached within 1–2 h after intake. Future capsule formulations are expected to reduce gastrointestinal toxicity like nausea, vomiting and diarrhoea, allowing more comfortable and safe administration.

Combination of oral paclitaxel with different oral cytotoxic therapies like vinorelbine or capecitabine or with cytostatic therapies like thyrosine kinase inhibitors has a clinical potential, but its safety and activity has to be addressed in future studies.

This study can serve as a template for the oral development of other drugs that show high affinity for ABC drug transporters and low and variable oral bioavailability.

In conclusion, this study revealed that weekly administration of oral paclitaxel, in combination with CsA, is an active and feasible treatment option for patients with advanced breast cancer who have received anthracycline-containing chemotherapy. Once daily dose administration with promising oral capsule formulations should lead to fewer adverse effects, better tolerance and compliance, without reduction in paclitaxel exposure and efficacy against solid malignancies. Current studies focus on testing of new capsule formulations of oral paclitaxel.

## Figures and Tables

**Table 1:** Patient characteristics

	**Patients (ITT)**		**Patients**	
**Character**	**All (*n*=29)**	**%**	**Evaluable (*n*=23)**	**%**
*Age (years)*				
Median (range)	50 (34–66)		48 (34–65)	
				
*Menopausal status*				
Premenopausal	1		1	
Postmenopausal	28		22	
				
*Hormonal receptor status*				
Positive	18	62		
Negative	9	31		
Unknown	2	7		
				
*Prior treatment*				
Chemotherapy	26	90	23	100
Anthracycline containing	25	86	23	
Antihormonal therapy	19	65	15	65
Radiotherapy	17	59	15	65
Surgery	14	48	12	52
				
*Previous anthracycline*				
Adjuvant	10	34	9	39
Palliative	15	52	14	61
				
*Prior chemotherapy lines*				
1	21	73	20	87
2	5	17	3	13
				
*Sites of metastases*				
Bones	20	69	16	70
Liver	18	62	15	65
Lymph nodes	16	55	12	52
Lungs	10	35	6	26
Contralateral breast	8	28	7	30
Skin	5	17	4	17
Pleura	4	14	3	13
Spleen	1	3	1	3
				
*No. of metastatic sites*				
Two or more	26	90	20	87
Three more	19	65	14	61
Four or more	6	21	4	17

ITT, intention to treat.

**Table 2:** Best response to weekly oral paclitaxel in combination with CsA (after up to 12 weekly administrations)

	**No. of patients (*N*=29)**	**% of evaluable patients (*N*=23)**	**% of ITT population (*N*=29)**
Complete response	0	0	0
Partial response	15	65.2	51.7
Stable disease	4	17.4	13.8
Progressive disease	4	17.4	13.8
Not evaluable	6		20.7

CsA, ciclosporin; ITT, intention to treat.

**Table 3:** Treatment toxicity (*N*=28)

	**1**	**2**	**3**	**4**	
**Grade**	***N* (%)**	***N* (%)**	***N* (%)**	***N* (%)**	**% Grades 3–4**
Leucocytopenia	0	5 (18)	13 (46)	2 (7)	53
Neutropenia	0	4 (14)	14 (50)	1 (4)	54
Thrombocytopenia	0	0	0	0	
Anaemia	2 (7)	7 (25)	0	0	
Alopecia	2 (7)	24 (86)			
Nausea	15 (57)	10 (36)			
Vomiting	17 (61)	4 (14)			
Fatigue	5 (18)	10 (36)	6 (21)		
Diarrhoea	13 (46)	6 (21)	1 (4)		
Neurotoxicity	11 (39)	5 (18)			
Arthralgia/myalgia	13 (46)	3 (11)			
Asthenia	3 (11)	11 (39)	1 (4)		
Abdominal cramps	6 (21)	5 (18)			
Constipation	10 (35)	1 (4)			
Nail changes	4 (14)	3 (11)			

Worst grade per patient. One patient was lost to follow-up after one administration.

**Table 4:** Pharmacokinetic parameters of twice on one day dosing of oral paclitaxel in 26 patients (data are listed as mean±s.d.)

	***C*_max_**			**AUC**			
	***μ*g l^−1^**	***μ*mol l^−1^**	***T*_max_ (h)**	***μ*g h l^−1^**	h*μmol l^−1^	***T*>0.1 *μ*mol l^−1^ (85.39 μg l^−1^) (h)**	***T*>0.05 *μ*mol l^−1^ (42.7 *μ*g l^−1^) (h)**
*Week 1*				4304 (1426)	5.04 (1.67)	13.2 (5.1)	23.8 (8.5)
Dose 1	333 (120)	0.39 (0.14)	1.51 (0.71)				
Dose 2	376 (94)	0.44 (0.11)	1.56 (1.50)				
							
*Week 2*				4005 (1110)	4.69 (1.3)	12.1 (3.8)	22.4 (7.5)
Dose 1	342 (102)	0.40 (0.12)	1.59 (0.80)				
Dose 2	376 (120)	0.44 (0.14)	1.29 (0.71)				

AUC=area under the plasma concentration–time curve.

The dose of paclitaxel was 90 mg m^−2^ and the dose of CsA was 10 mg kg^−1^.
